# Challenges and Practices in Building and Implementing Biosafety and Biosecurity Programs to Enable Basic and Translational Research with Select Agents

**DOI:** 10.4172/2157-2526.S3-015

**Published:** 2013-04-29

**Authors:** Colleen B. Jonsson, Kelly Stefano Cole, Chad J. Roy, David S. Perlin, Gerald Byrne, members of the RBL-NBL Directors Network

**Affiliations:** 1 Department of Microbiology and Immunology, University of Louisville, KY, USA; 2 Center for Predictive Medicine for Biodefense and Emerging Infectious Diseases, University of Louisville, KY, USA; 3 Center for Vaccine Research, Regional Biocontainment Laboratory, University of Pittsburgh School of Medicine, Pittsburgh, PA, USA; 4 Department of Immunology, University of Pittsburgh School of Medicine, Pittsburgh, PA, USA; 5 Tulane National Primate Research Center, Regional Biosafety Laboratory, Covington, LA, USA; 6 Division of Microbiology and Immunology, Tulane University School of Medicine, New Orleans, LA, USA; 7 Public Health Research Institute and UMDNJ Regional Biocontainment Laboratory, New Jersey Medical School-UMDNJ, Newark, NJ, USA; 8 Department of Microbiology and Immunology, University of Tennessee, Memphis, TN, USA

## Abstract

Select agent research in the United States must meet federally-mandated biological surety guidelines and rules which are comprised of two main components: biosecurity and biosafety. Biosecurity is the process employed for ensuring biological agents are properly safeguarded against theft, loss, diversion, unauthorized access or use/release. Biosafety is those processes that ensure that operations with such agents are conducted in a safe, secure and reliable manner. As such, a biological surety program is generally concerned with biological agents that present high risk for adverse medical and/or agricultural consequences upon release outside of proper containment. The U.S. Regional and National Biocontainment Laboratories (RBL, NBL) represent expertise in this type of research, and are actively engaged in the development of programs to address these critical needs and federal requirements. While this comprises an ongoing activity for the RBLs, NBLs and other facilities that handle select agents as new guidelines and regulations are implemented, the present article is written with the goal of presenting a simplified yet comprehensive review of these requirements. Herein, we discuss the requirements and the various activities that the RBL/NBL programs have implemented to achieve these metrics set forth by various agencies within the U.S. Federal government.

## Introduction

The goal of the present article is to provide a cohesive summary of administrative and operational approaches that can be used in building and subsequent management of research with select agents within biosafety level-3 laboratories (BSL-3). Select agents and toxins are biological pathogens or derivatives, respectively that have the potential to pose a severe threat to human, animal, or plant health [1– 3]. The Regional and National Biocontainment laboratory (RBL/NBL) consortium has the experience and mandate to provide leadership in this area and herein we discuss our shared experiences to achieve the present (and anticipated future) metrics set forth by the various government agencies for research with select agents in our respective institutions. Although there are several possible paths to achieving regulatory compliance, these suggestions are based on the successful practices used by our facilities. First, we summarize the history with respect to the laws and guidelines for possession, use or transfer of these agents in the past two decades.

The Antiterrorism and Effective Death Penalty Act of 1996, Public Law104-32, authorized the Secretary of Health and Human Services to establish and enforce safety procedures for the transfer of biological or what is now commonly referred to as “select” agents. In section 511, the law states that the transfer and possession of potentially hazardous biological agents should be regulated to protect public health and safety, but ensure that individuals and groups with legitimate objectives have access for clinical and research purposes [3]. Further, the law requires safeguards to prevent access to select agents by domestic or international terrorists. Shortly after the terrorist attacks on September 11 and the anthrax mailings in 2001, the USA PATRIOT Act (Public Health 107- 56-Oct. 26, 2001) and the Public Health Security and Bioterrorism Preparedness and Response Act of 2002 (Public Health 107-188- June 12, 2002) strengthened the requirements for accountability and restrictions for access to agents on the select agent list.

The Centers for Disease Control and Prevention (CDC) and the U.S. Departments of Agriculture (USDA)-Animal and Plant Health Inspection Service (APHIS) implement the provisions of Public Law 107–188 through a series of regulations that culminated in two notices in the Federal Register. The first, published in the Federal Register by the U.S. Department of Health and Human Services (HHS) is the 42 Code of Federal Regulations (CFR) “Possession, Use, and Transfer of Select Agents and Toxins; Final Rule” [4–8]. The second, also published in the Federal Register by USDA-APHIS, is the 7 CFR Part 331 and 9 CFR, Part 112 “Agricultural Bioterrorism Protection Act of 2002; Biennial Review and Republication of the Select Agent and Toxin List; Amendments to the Select Agent and Toxin Regulations; Final Rule”. HHS regulates those agents that might affect public health and safety whereas USDA regulates those agents that may affect animal and plant health and products. Overlap agents are those regulated by both HHS and USDA. Recently, these published final rules were revised and published in the Federal Register (October 5, 2012) as part of a biennial review. The National Select Agent Registry Program (NSARP) is the primary group within the federal government that oversees the possession and activities with select agents (http://www.selectagents.gov/). NSARP requires facilities to register if they possess, use or transfer biological agents and toxins that pose a significant threat to public, animal or plant health, or animal or plant products. Once registered, individuals cleared by the US Department of Justice may have access to select agents at that institution. Hence it is critical that each institution engaging in research involving select agents develop strong, robust programs that meet the needs of current and anticipated future regulations.

The National Select Agent Program is jointly comprised of the CDC/Division of Select Agents and Toxins and the APHIS/Agricultural Select Agent Program. The NSARP requires a Responsible Official (RO) to be designated at each institution. The RO is the official designee in the eyes of the NSARP for a particular entity, and as such is ultimately responsible for the appropriate adjudication of an institution’s Select Agent program. In registration of a facility for select agents, a number of factors are required which are detailed in table 1. Importantly, during inspections by the CDC and USDA following an application for select agent registration, a registrant entity must demonstrate compliance in four major areas (Table 1).

These federal requirements may include those mentioned previously as well as policies developed by other agencies such as the Department of Defense (DoD) directives 5210.88 “Safeguarding Biological Select Agents and Toxins”, 5210.89 “Minimum Security Standards for Safeguarding Biological Select Agents and Toxins”, and Army Regulation, AR 50-1, “Nuclear and Chemical Weapons and Materiel, Biological Surety” [9]. Institutions funded by these agencies and DoD are required to comply with these regulations.

The terrorist attacks on September 11 and the anthrax mailings in 2001 [10] set into motion a Blue Ribbon Panel of biodefense experts convened by the National Institute of Allergy and Infectious Diseases (NIAID), a component of the National Institutes of Health (NIH). These discussions described the critical need for specialized facilities to conduct academic research with biodefense and emerging infectious disease agents; many of which are select agents. Collectively, federal construction grants for 13 Regional (RBL) and 2 National Biocontainment (NBL) Laboratories were awarded. Each of the completed facilities have developed unique research focal areas within bacteriology and virology requiring various levels of containment (Table 2) and technical capabilities (Table 3), while all share common operational capabilities. While all of the facilities have small animal capability, several RBLs (University of Pittsburg, Tulane University and George Mason University) and both NBLs also have the ability to conduct nonhuman primate research. The RBL and NBL network was also originally envisioned as a regional resource to assist national, state, and local public health efforts in the event of a bioterrorism or infectious disease emergency. These facilities were constructed with the recognition that new and revised rules and regulations for biosecurity are a continual and dynamic process of various governmental agencies, the outcomes of which affect the access, handling, and transport of over 80 listed select agents [11–13].

## Development of Principles and Practices of Security, Accountability, Personnel Responsibility, Worker and Community Health and Safety

In the development of an institution’s select agent program, interdisciplinary, intra-institutional, cooperation and discussions across academic and operational groups are critical to build a framework for regulation and oversight of people and practices. These discussions include how to approach the initial hiring process of individuals that will work in the facility with select agents. For example, is the probationary period sufficient to gauge a person’s ability or willingness to follow regulations? How will the training program, the development of the standardized operating procedures (SOPs) for storage/use/transfer, and emergency response be designed, developed and managed for compliance and adherence? What are the research programs within the institution and how will they be regulated to meet federal requirements for work with select agents [14]? In general, taken together the federal guidelines mandate that an institution’s select agent program addresses the following areas to be considered successful: (1) security (physical and information), (2) accountability, (3) personnel responsibility or reliability, and (4) worker & community health and safety. Approaches in each of these areas are discussed in the following. In the most recent update of 42 CFR Part 73, select agents have been further classified as Tier 1 or not [5]. Tier 1 regulations which require higher levels of security and guidelines not previously mandated (personal reliability) will also be discussed. HHS/CDC have designated Tier 1 agents as those select agents and toxins that “present the greatest risk of deliberate misuse with the most significant potential for mass casualties or devastating effects to the economy, critical infrastructure, or public confidence”.

## Physical security

At a minimum, the physical security system at non-Tier 1 institutions must be capable of completely and physically securing the laboratory area where select agents are stored and alerting local security personnel and/or university and local law enforcement agency if this physical security system is breached. Current Tier 1 select agent regulations require at least three layers of security for safe guarding of select agents and materials. Specifically, ‘‘entities possessing Tier 1 select agents and toxins must have a minimum of three security barriers where each security barrier adds to the delay in reaching secured areas where select agents and toxins are used or stored” [5]. A typical layered security approach may include (1) a perimeter (e.g., fence, ballards) with controlled access points, (2) locked building entrances with electronic access control and elevator/stairwell access control, (3) additional access control at the entrance to the laboratory space and (4) locks on the individual biological agent storage units (ultra-low temperature or liquid nitrogen freezers) or doors to those areas. A layered system that tracks individual access events (e.g., through the use of biometric scanners and unique key cards), and incorporates video surveillance cameras in key locations is a highly useful tool to meet expectations for continuous facility monitoring (required for Tier 1).Security systems also incorporate back-up battery power supply to maintain access control in the event of power outage. More sophisticated facilities may have emergency generators for back-up emergency power. Additional considerations for preclinical research include approaches for safe guarding of records and monitoring of equipment and environment as mandated by good laboratory and/or manufacturing practices (GLP, GMP) [15].

The administrative aspect of security programs are based on a rubric of a threat risk assessment which details potential threats, the facility’s relative vulnerability and perceived risk to each threat, and ways in which to mitigate potential threat risks [13]. The administrative security program provides documentation of personnel security access levels and incorporates a method for investigation and documentation of any security-related events (e.g., alarms, unauthorized access). Also included in the Biosecurity, Emergency and Incident Response Plans is an additional plan detailing procedures that are followed to maintain laboratory security integrity in the event of disaster (e.g. natural disaster, fire, sabotage, infrastructure failure, biological/chemical spills, workplace violence, theft, etc.). The program also details internal and external reporting procedures for security breaches (subject to applicable governmental regulations).

In general, a risk-based, layered approach encompasses the collective needs of individuals that require access to specific select agent areas, equipment, information technology and research information. Typically this includes access for people that reside in individual research laboratories, administrative and core facility faculty and staff, operating engineers, institutional biosafety personnel, cleaning and maintenance crews, and the local and institutional emergency response personnel. Normal and emergency response design and operating conditions are of importance in development of the biosecurity plans. Planned response to equipment and system failures are required of all persons working in high containment. Operational scenarios and programmatic steps that ensure that a proper approach has been developed to safeguard select agent materials in the event of an outage are critical to any entity. Biosecurity plans are continuously re-evaluated at regular intervals to ensure compliance with current regulations; routine exercises and/or drills are conducted to demonstrate the feasibility and validity of plans with all personnel involved in the operation, maintenance, and use of these facilities.

Finally, it is important that the physical security of the building is maintained by emergency power that is a combination of centralized and distributed backup power sources in order to ensure that critical systems continue to function as designed even in the event of a loss of power. The design of the critical back-up systems require that emergency power is supplied for a period of time that is well in excess of what is necessary for the notification and arrival of law enforcement and emergency response personnel.

## Information security: design and implementation of select agent tracking software

One substantial investment in time for establishment of select agent research program is a thorough understanding of the record-keeping requirements. The NSARP requires that each institution maintain a secure database of the select agent pathogens that are stored on site. While written data logs are acceptable, software programs, including those that will implement bar code facilitated tracking to track all select agent samples and ancillary data (Table 4) as required by the CDC, would be a valued asset. The challenge lies in the identification of software tracking programs capable of being tailored to the needs of each research program and select agent accountability. In 42 CFR Part 73.17 (1), 7 CFR Part 331.17(1) and 9 CFR Part 121.17(1), the regulations include the requirement for an accurate inventory, which is defined as an, “Accurate, current inventory for each select agent (including viral genetic elements, recombinant nucleic acids, and recombinant organisms) held in long-term storage (placement in a system designed to ensure viability for future use, such as a freezer or lyophilized materials)”. In addition to the above database information, any material determined to be long term storage must be maintained in a secure location and detailed, accurate records must be kept. If one has conducted a GLP protocol with a select agent, then both GLP and select agent tracking and storage guidelines and regulations are required, which increase the regulatory complexity of the activity [15]. While materials determined to not be long term storage do not require detailed, accurate records, the entity must still have mechanisms in place to control the distribution of the material and to track the creation of the working material from long term storage materials. Hence, institutions will be required to provide records, if requested, that document the stock source of all production quantities of agents. Further, institutions must have protocols in place and be able to produce documentation for the transfer and accountability of inventories when the investigator responsible for the inventory departs the entity as a result of change in employment, retirement, death, sabbatical, or other reasons for no longer having an active role in the entity. Finally, a software tracking program must have the flexibility to be modified as new federal rules and guidelines are developed.

## Accountability

A system of accountability is absolutely essential to maintaining biological surety. One important element is the inventory of all select agents. Select agent inventories, an important element of biological surety, is required to provide information with regard to agent identity, source, date of receipt, number of vials, and volumes for each stock held at a particular site. The inventory also facilitates tracking personnel access to each agent and is a permanent record of the addition and removal of vials from each stock. This inventory may be maintained electronically on a secured computer or server, managed by customized software at many facilities. However, physical inventories are required as a result of federal rules and are checked on a quarterly basis by the entity’s RO/ARO for accountability.

A second aspect of accountability is shipment and receipt records of select agents and the corresponding SOP detailing critical aspects of this process. The SOP provides guidance on the process for obtaining any necessary federal permits for transfer or import/export as well as proper shipment packaging and documentation practices for select agents. All shipment tracking documentation is maintained to ensure continuity and coordination among the sender and receiver and the Federal agency (NSARP) overseeing the transfer. Importantly, shipment loss or package damage is required to be reported immediately to appropriate personnel/authorities according to standard procedures. The CDC (NSARP) is the primary contact agency within the United States. However, it may also be necessary to contact local, state and federal agencies depending on the circumstances surrounding the loss (i.e., theft). Employees working within select agent laboratories are trained to comprehend the reporting structure in the event of a theft, loss, or release of agent; facility training covers this reporting structure. Losses involving select agents are reported to the entity’s RO; he/she is the responsible party for contacting the CDC within 24 hours of the loss as well as for following up with an investigation of the event and subsequent notification to local authorities as applicable about the loss of agent.

## Personnel responsibility

The effectiveness of a laboratory’s biological surety (or personnel reliability) program ultimately lies with those who have access to and use the select agents. A policy of personnel responsibility assures that workers with access to select agents are not impaired and do not pose a risk of inappropriate behavior. Policies relating to personnel responsibility are clearly documented and communicated to all staff members prior to initiation of work in the biocontainment laboratory environment. Disciplinary action resulting from noncompliance must be outlined in the institution’s policy; these types of policies may reside within the unit having oversight of the laboratory in a University setting.

An effective personnel responsibility policy is composed of a worker prescreening process as well as ongoing monitoring for the term of facility employment. Prescreening includes initial screening for criminal history and is required by the Department of Justice prior to registration of an individual as a user of Select Agents. Individuals with prior criminal records will not be allowed access to select agents under US law. While criminal record checks are one aspect of personnel reliability, all persons having access to select agent laboratories should be cognizant of abnormal behavior patterns (e.g., aggressiveness, violence, depression, high stress or suspicious behavior) that may be harbingers of unallowable addictive behaviors (e.g., substance abuse) within these occupational environments.

## Worker and community health and safety

A health and safety program assures that laboratory activities have acceptable levels of risk for the workforce and community. Laboratory personnel are enrolled in medical surveillance programs that are commensurate with risk involved in working with specific Select Agents. These medical monitoring programs provide screening upon hire and ongoing surveillance to ensure that laboratory staff is immunized and maintain their health status to perform their duties within containment. Some preexisting medical conditions preclude individuals from certain types of laboratory work.

All employees wearing respiratory protection must be enrolled in a respiratory protection program. Personnel, who wear N-95 respirators, must be fit tested at least annually by an approved person prior to working in a biocontainment facility. Individuals who wear Powered Air Purifying Respirator (PAPR) must be trained initially on the use and care of the PAPR. Annual training may be required for this type of respirator. As PAPR blowers can interfere with hearing and communication, this should be considered in the training of employees and students.

In addition to personal protective equipment (PPE) required for the agents in use, engineering controls will be utilized whenever possible to protect workers and the community from biohazards. A primary containment unit such as a biological safety cabinet (BSC) will be used when handling certain biological agents to prevent the escape of aerosols into the environment and/or containment of certain equipment [16]. The type of BSC used depends on the associated hazard in order to protect workers and the environment effectively. Other engineering controls that are in place in the BSL-3 include high efficiency particulate air (HEPA) filtration and/or negative pressure, self-contained caging, often with individual air purifying systems used to eliminate laboratory exposures of pathogens from infected animals [17,18]. Additional programmatic requirements pertaining to the use of Select Agents in animals has been recently published [17]. Specialized tubes with bioseal closures and specialized housing are used to avoid aerosol generation from centrifuges and other mechanical equipment.

Select agent laboratories are required to have a biosafety manual and accompanying SOPs detailing safety practices and procedures in addition to standard practices (e.g., (1) Occupational Safety and Health Administration (OSHA) blood borne pathogens, chemical and hazardous waste handling, emergency contact information). If space allows, computers may be made available in each laboratory which enable electronic access to these various SOPs and protocols. Each principle investigator is also responsible for ensuring his/her personnel follow all procedures as outlined in the approved biosafety manual. Spot-checking and post-training monitoring is conducted to ensure full compliance with SOPs. Retraining is mandated for individuals who deviate from approved protocols. The employee health and safety department or equivalent at each entity, typically the RO and/or ARO, and the facility director/manager should be actively involved in the annual review of all BSL-3/ABSL-3 Biosafety Manuals and unique BSL-3 and -4 lab operation SOPs such as entry and exit. All researchers must follow the practices and procedures for biosafety and biosecurity as published by the CDC, NIH, USDA-APHIS, and the institution. Additional information pertaining to medical surveillance of researchers with access to select agents and toxins must be made readily available to all personnel.

## Intensive biosafety and biosecurity training for faculty and staff for select agents

Several multi-day or week-long intensive professionally directed courses are available for those engaged in select agent or BSL-3 and BSL-4 research. Topics typically covered include the CDC Biosafety in Microbiological and Biomedical Laboratories (BMBL), NIH Recombinant DNA Guidelines [19], Biosafety committees, other administration aspects, risk assessment, select agent regulations and administration, HEPA filters and biosafety cabinet certification and introductory BSL-3 or BSL-4. These courses provide faculty and staff with an overall knowledge of BSL-3 or BSL-4 research, and provide the ability of such individuals to move forward as trainers in a “Train the Trainer” program (the latter being vital to ensuring the implementation of safety and security of the select agent program). However each facility is different and each program will have unique aspects depending upon their research focus and the select agent program must necessarily create processes that facilitate and safeguard research and demonstrate competency [20].

Each institution with a select agent program must develop integrated and comprehensive training programs in collaboration with the Environmental Health and Safety department (EH&S). Required research training may include guidelines set forth by OSHA, Environmental Protection Agency (EPA), NIH, CDC, USDA APHIS, U.S. Food & Drug Administration (FDA), National Fire Protection Agency (NFPA), and/or World Health Organization (WHO). The standard select agent training program may include lecture and/or web-based as well as hands-on training activities for (1) personal protective equipment, (2) blood borne pathogens, (3) BSL-2, BSL- 3, and/or BSL-4 laboratory principles and practices, (4) principles of biosecurity, (5) agent specific training and (6) bioethics and dual use training. Additionally, facilities engaged in translational research may also consider modules in (1) GLP, (2) data management and sharing, (3) quality control management, and (4) interpretation of results and reporting. For those labs engaged in specimen collection, training modules may include: (1) principles of safe collection, transport, inventory, tracking and storage of specimens, (2) how to package and ship specimens, (3) dangerous goods shipping, and (4) inactivation of biological materials for use in BSL-2 research. All training should be documented and updated at least annually.

## Management and implementation of training matrices

The select agent training programs are designed with people, facilities and processes in mind. Implementation of this type of program may benefit from a phased approach, which maximizes participation, yet is cost effective. The successful management and implementation of such a large and complex training effort requires efforts along three lines: (1) assessment and refinement, (2) implementation, and (3) development of sustainment and a culture of compliance (Table 5).

## Proficiency in practices and techniques

One person should have primary oversight and responsibility for ensuring that personnel have a track record and/or demonstrate proficiency in standard microbiological practices and techniques before working with select agents. Generally the facility director/manager or the biosafety officer will be the designated individual. Proficiencies include prior experience in handling human pathogens or cell cultures. Responsibilities include a specific training program provided by a staff scientist proficient in safe microbiological practices and techniques. Trainees should show familiarity with practices and policies detailed in the biosafety manual and are knowledgeable of the potential health hazards of working with the organisms under study. Trainees must also demonstrate proper use and maintenance of research equipment such as the BSC or tissue digestor [21]. The supervisor will be responsible for observing trainees to ensure that personnel have appropriate knowledge of all research protocols and are able to demonstrate proper execution of these protocols and use of equipment prior to approval. This approach is also applicable to research conducted under GLP.

## Training for authorized users

Only authorized lab users will be granted access to biocontainment facilities where select agents are used and/or stored. All individuals who are listed as having access to select agents and toxins must complete biosafety, security and incident response training on at least an annual basis [22]. Biosafety training includes information about the infectious agents that are stored and used by the entity, including health considerations posed by the Risk Groups 3 agents, routes of exposure, symptoms, medical surveillance, PPE usage, incident response, post-incident response, post-exposure medical surveillance, post-exposure reporting and follow-up procedures. In addition, personnel should be trained on facility specific equipment, controls for facility and biosafety practices and procedures. The level and detail of training will reflect the investigator’s research goals. As changes occur in tasks or procedures that may affect the employee’s exposure, additional training should be provided. As part of initial and refresher training, all personnel must review and sign laboratory manuals to acknowledge their existence and location as needed for consultation of procedural or safety concerns, and for review of any updated material. By signing, personnel acknowledge that they have been advised of the hazards associated with this research, that they have been properly trained in the handling of the biological agent following BSL-3 guidelines, and that they agree to adhere to these regulations.

Prior to beginning work in a BSL-3 or BSL-4 laboratory, it is mandatory that individuals have documented experience working in similar laboratory settings at a lower biosafety level in that area of research. Examples of documented training that may be required include: Laboratory Safety Training, Hazardous Waste Training, Respiratory Protection Training, Bloodborne Pathogens Training, Radiation Safety Training, BSL-3 Training and Agent-Specific Training. Other training programs, including observation of work at BSL-2 using BSL-3 practices, may be required at the discretion of the EH&S office prior to working at BSL-3 with select agents. In addition to those guidelines published by the CDC in the BMBL 5th Edition (available on-line and in print), BSL-4 laboratories have jointly published standardization of training and potential opportunities [23–25]. Additional papers have been published for BSL-3 laboratories, which provide details of the biosafety and decontamination across numerous topics that may be considered in implementation of programs and their annual review [22,26–32].

## Tabletop exercises

Tabletop exercises are training methods that is employed to enhance emergency preparedness and response by assessing readiness for unexpected events. In most facilities, a typical planned exercise involves a staged emergency situation in which laboratory personnel, building support (e.g., physical plant), biosafety, building security, public safety, local, state and federal law enforcement, EMS, fire and hazmat teams participate and respond as if a real emergency has taken place [33]. The establishment of incident command posts and authority for meeting medical needs of individuals; securing the facility and protecting the public are all tested. At the end of the exercise, all participants attend a review session from designated evaluators who critique the response and make recommendations for improved communication and efficiency of the overall management of the incident. Participants discuss details of management issues, roles and responsibilities, communication and coordination, and mobilization of resources, and discussions of SOPs and policies. Finally, recommendations are made to all stakeholders.

## Web-based training

One mechanism for training can use web-based approaches. Web-based training generally contains visual and/or audio based material followed by a quiz to determine that personnel learned and retained material. While initial training programs are often lecture-based, standard and routine material, updates and retraining of employees may be useful in web-based formats to minimize the time for both the educator and the employees. Web-based coursework and quizzes are useful for retraining of individuals who have breached policies and procedures. Training may last 30 minutes per focal topic and would be followed by a web based examination to assess retention. Over the long term, this approach reduces cost and provides more individualized training for personnel on biosafety and biosecurity concepts.

## Hands-on training

A second and probably more critical component of the training program is the hands-on training. This provides time for the trainer to evaluate the trainee and his/her ability and comfort level with biosafety and biosecurity principles and practices for competency [20]. As biosafety and biosecurity risks increase, the trainer to trainee ratio should ideally decrease; particularly when the concepts require reasoning and independent thought in a hazardous environment. Ideally, in a university setting, the head of the lab or a senior person in the lab provides the hands-on training. The smaller student-teacher approach stresses thinking and reasoning approaches rather than rote memorization and focuses on the risks at hand. This is especially important for basic research, which is not a static process. The instruction provided should require productive behavior and/ or appropriate feedback. Each hands-on training session should be documented as to time and tasks covered with space for comments and concerns noted. The amount of time or number of sessions required for hands-on training will vary per trainee based on the individual’s prior level of experience and the institution’s policy.

## Compliance

A continuous, on-the-job biosafety (protecting the people from pathogens) and biosecurity (protecting the pathogens from people) program is essential to maintain safety awareness and compliance among laboratory and support staff. Laboratory core staff, with the assistance of the EH&S biosafety staff, play a key role in “buy-in”, staff training and awareness. The effectiveness of biosafety and biosecurity training depends on management commitment, motivational factors, adequate initial job training, good communications, and ultimately being a part of the organization’s goals and objectives (Laboratory Biosafety Manual, Third Edition, WHO, 2004; Biorisk Management: Laboratory Biosecurity Guidance, WHO, 2006; Laboratory Biorisk Management Standard, CWA 15793:2008). Clearly, compliance is a team effort and it is important to stress early in the training process that all personnel must not only rely on each other for support, but must be willing and able to report problems and concerns to their supervisor without risk of punitive action.

## Conclusion

### Implementation of regulatory requirements for work with select agents

The goal of this article is to present a cohesive operational approach to provide guidelines for those who work with biological select agents. While mandated, the regulations allow much discretion about what constitutes proper compliance and operational integrity. The RBL/NBL consortium of labs has the experience to provide guidance for building and implementation of administrative and operational programs and practices that can be further adapted to other labs outside the network. While each laboratory may have unique circumstances that may result in slightly different implementation, the current document sets forth guidance on the larger issues that need to be addressed. Creating a culture of regulatory compliance will lead to safer and more efficient operations to ensure the safety and reliability of systems for both our scientist and the public as we safeguard against security breaches that could threaten public safety.

Registration of each select agent laboratory, permits for obtaining new select agents (programmatically or emerging), on-going training and regulatory compliance requires substantial investment of administrative resources to maintain effective research programs, especially those involved in translational endeavors. On-going training and record-keeping requirements are key regulatory challenges that PIs and their trainees face in working with these agents, which necessarily slow the pace at which research is conducted. Specialized governmental and institutional support is critical for those engaged in such highly regulated programs involved in the discovery of new antivirals, therapeutics, vaccines and diagnostics for biodefense and emerging pathogens.

## Figures and Tables

**Table 1:** Essential programmatic components of a select agent laboratory.

Guideline	Goal
Ensure the accuracy of the records and databases	Inventories must be maintained for 3 years for agents, access to areas where agents are stored, transfers, training and all records pertaining to select agent (or toxins). Each agent that is housed must be registered and each individual must have DOJ approval for access.
Develop safety, security and incidence response plans	The security plan must be based on a site-based risk assessment that safeguards the theft, loss or release of the agent. Incidence response plans must be in compliance with local emergency responders and available to employees.
Conduct drills or exercises to test effectiveness of the plans/ conduct safety and security training	Registered entities must conduct drills, review plans, conduct safety and security training, inspections, BSC certification, and verification of all operational parameters (e.g., BSL3, verification of facility design). The institution must conduct safety and security training that includes risks and hazards and is tailored to the needs of the individuals involved.
Immediate notification of theft, loss or release	As defined by the regulations, these three areas require notification internally and externally (CDC). This occurs when the agent is released accidentally outside of the primary containment barrier due to a failure in the barrier, an accidental spill or an unauthorized removal of the agent.

**Table 2:** Institutional regulatory capability.

Location Institution*	BSL-3	BSL-3+	BSL-4	Select Agent	Tier I	GLP≠	GMP≠
Duke U Human Vaccine Institute Regional Biocontainment Laboratory (RBL)	Y	Y	N	Y	Y	Y	N
Colorado State University RBL	Y	Y	N	Y	Y	Limited	Y
University of Pittsburgh, Center for Vaccine Research RBL	Y	Y	N	Y	Y	N	N
University of Alabama Birmingham Southeast Biosafety Laboratory Alabama (SEBLAB)	Y	N	N	Y	Y	N	N
University of Chicago The Howard T. Ricketts Laboratory (HTRL)	Y	Y	N	Y	Y	N	N
University of Medicine and Dentistry of New Jersey RBL	Y	N	N	Y	Y	Y	N
University of Missouri-Columbia RBL	Y	N	N	Y	Y	N	N
Tufts University/New England RBL (NE-RBL)	Y	N	N	Y	?	N	N
Tulane National Primate Research Center RBL	Y	Y	N	Y	Y	N	N
University of Tennessee Health Science Center RBL	Y	N	N	Y	Y	Y	N
University of Louisville Center for Predictive Medicine RBL	Y	Y	N	Y	Y	Y	Limited
George Mason University/Biomedical Research Laboratory (BRL)	Y	N	N	Y	Y	Y	N
University of Texas Medical Branch at Galveston/National Laboratory (GNL)	Y	Y	Y	Y	Y	Y	N
Boston University# National Emerging Infectious Diseases Laboratory (NEIDL)#	Y	Y	Y	Y	Y	N	N

* At the writing of this review The University of Hawaii RBL had not yet begun construction.

# The Boston University NEIDL had been completed and occupied at BSL2; date for occupancy at BSL3/4 was pending.

≠ GLP-good laboratory practice, good manufacturing practice

**Table 3:** Institutional technical capability.

Institution	HTS	Whole Body Imaging	Live Cell Microscopy	Aerosol	Preclinical Testing	Hematol	Flow Cytometry	Live Cell Sorting
Duke U Human Vaccine Institute Regional Biocontainment Laboratory (RBL)	N	IVIS	N	Y	Y	Y	Y	Y
Colorado State University RBL	N	IVIS	N	Y	Y	N	Y	N
University of Pittsburgh, Center for Vaccine Research RBL	N	IVIS, CT/PET (NHP)	Y	Y	Y	Y	Y	Y
University of Alabama Birmingham Southeast Biosafety Laboratory Alabama (SEBLAB)	N	N	N	Y	Y	N	N	N
University of Chicago The Howard T. Ricketts Laboratory (HTRL)	N	N	N	Y	N	Y	Y	Y
University of Medicine and Dentistry of New Jersey RBL	N	IVIS	N	Y	Y	Y	Y	N
University of Missouri-Columbia RBL	N	N	N	N	N	N	N	N
Tufts University/New England RBL (NE-RBL)	N	N	N	N	Y	N	N	N
Tulane National Primate Research Center RBL	N	N	N	Y	Y	Y	Y	Y
University of Tennessee Health Science Center RBL	Y	IVIS	Y	Y	Y	Y	Y	N
University of Louisville Center for Predictive Medicine RBL	Y	IVIS CT/PET/ SPECT	Y	N	Y	Y	Y	Y
George Mason University/ Biomedical Research Laboratory (BRL)	Y	IVIS	N	N	Y	Y	Y	Y
University of Texas Medical Branch at Galveston/National Laboratory (GNL)	Y	IVIS, CT/PET	N	Y	Y	Y	Y	Y
Boston University^#^ National Emerging Infectious Diseases Laboratory (NEIDL)^#^	N	IVIS, CT/SPECTMRI	Y	Y	Y	Y	Y	Y

**Table 4:** Management and implementation of training matrices for select agent research.

Assessment and refinement phase	Working with Principle Investigators, the facility management and EH&S team will assess the personnel, facilities, and pathogens to identify and fill the facility administration and operational gaps and drive the training matrix accordingly. Initial and ongoing assessment of technical capability, procedures and practices will be assessed by a team composed of the Biosafety Specialist, the PI and key staff (e.g., vivarium).
Implementation	Beginning with the end in mind, it will be important to create momentum and enthusiasm for the program, and every opportunity should be used to encourage cooperation. In general, lecture and laboratory-based training can be limited to groups of 20–25 and 4–6, respectively. Overall, it will be important to be flexible to ensure maximal participation and opportunities for clarification of questions.
Development of sustainment and compliance	To develop organic capability and provide sustainment to the training matrices that is cost effective and flexible, web-based and hands-on training is essential.

**Table 5:** Select agent tracking and record keeping.

Component to track	Specific example
Pathogen name and characteristics	Strain designation, GenBank Accession number
Quantity acquired from another individual or entity and its expansion	Containers, vials, tubes, etc., date of acquisition, and the source (name, institution).
Location stored	Building, room, freezer, shelf, box
When moved from storage	By whom and when returned to storage and by whom; the select agent used and purpose of use
Records created under Section 16 of 7 CFR Part 331, 9 CFR Part 121, and 42 CFR Part 73 (Transfers)	For intra-entity transfers (sender and the recipient are covered by the same certificate of registration), the select agent, the quantity transferred, the date of transfer, the sender, and the recipient; Records created under Section 19 of 7 CFR Part 331, 9 CFR Part 121, and 42 CFR Part 73 (Notification of theft, loss, or release)”
